# Mechanisms of Rapid Reactive Oxygen Species Generation in Response to Cytosolic Ca^2+^ or Zn^2+^ Loads in Cortical Neurons

**DOI:** 10.1371/journal.pone.0083347

**Published:** 2013-12-10

**Authors:** Aaron Clausen, Taylor McClanahan, Sung G. Ji, John H. Weiss

**Affiliations:** 1 Department of Neurology, University of California Irvine, Irvine, California, United States of America; 2 Department of Anatomy and Neurobiology, University of California Irvine, Irvine, California, United States of America; Karolinska Institutet, Sweden

## Abstract

Excessive “excitotoxic” accumulation of Ca^2+^ and Zn^2+^ within neurons contributes to neurodegeneration in pathological conditions including ischemia. Putative early targets of these ions, both of which are linked to increased reactive oxygen species (ROS) generation, are mitochondria and the cytosolic enzyme, NADPH oxidase (NOX). The present study uses primary cortical neuronal cultures to examine respective contributions of mitochondria and NOX to ROS generation in response to Ca^2+^ or Zn^2+^ loading. Induction of rapid cytosolic accumulation of either Ca^2+^ (via NMDA exposure) or Zn^2+^ (via Zn^2+^/Pyrithione exposure in 0 Ca^2+^) caused sharp cytosolic rises in these ions, as well as a strong and rapid increase in ROS generation. Inhibition of NOX activation significantly reduced the Ca^2+^-induced ROS production with little effect on the Zn^2+^- triggered ROS generation. Conversely, dissipation of the mitochondrial electrochemical gradient increased the cytosolic Ca^2+^ or Zn^2+^ rises caused by these exposures, consistent with inhibition of mitochondrial uptake of these ions. However, such disruption of mitochondrial function markedly suppressed the Zn^2+^-triggered ROS, while partially attenuating the Ca^2+^-triggered ROS. Furthermore, block of the mitochondrial Ca^2+^ uniporter (MCU), through which Zn^2+^ as well as Ca^2+^ can enter the mitochondrial matrix, substantially diminished Zn^2+^ triggered ROS production, suggesting that the ROS generation occurs specifically in response to Zn^2+^ entry into mitochondria. Finally, in the presence of the sulfhydryl-oxidizing agent 2,2'-dithiodipyridine, which impairs Zn^2+^ binding to cytosolic metalloproteins, far lower Zn^2+^ exposures were able to induce mitochondrial Zn^2+^ uptake and consequent ROS generation. Thus, whereas rapid acute accumulation of Zn^2+^ and Ca^2+^ each can trigger injurious ROS generation, Zn^2+^ entry into mitochondria via the MCU may do so with particular potency. This may be of particular relevance to conditions like ischemia in which cytosolic Zn^2+^ buffering is impaired due to acidosis and oxidative stress.

## Introduction

Excessive glutamate release and overactivation of glutamate receptors (“excitotoxicity”) contributes to neuronal injury in conditions including stroke, prolonged seizures and trauma. Although many events come into play at different stages of the injury process, generation of reactive oxygen species (ROS) may be an important early contributor. A key trigger of the injury has been widely considered to be rapid Ca^2+^ influx through highly Ca^2+^ permeable N-Methyl-D-aspartic acid (NMDA) receptors. Mitochondria, which can take up and buffer large cytosolic Ca^2+^ loads, have long been considered to be critical targets of the Ca^2+^ loads, with a number of studies finding NMDA receptor mediated Ca^2+^ rises to result in release of ROS from the mitochondria into the cytosol [1-3]. Another mechanism through which excitotoxic Ca^2+^ overload may mediate injury is via activation of NADPH oxidase (NOX), a multi-subunit cytosolic enzyme that functions as a transmembrane electron transporter and produces superoxide by reducing molecular oxygen. Indeed, a recent study suggests that NOX translocation and activation may predominate as a mechanism of ROS generation during excitotoxic NMDA exposure [4]. 

 Large amounts of Zn^2+^ are present in the brain, but free Zn^2+^ levels are normally extremely low. However, observations that Zn^2+^ accumulates in many degenerating neurons after ischemia or prolonged seizures, and that its chelation decreases resultant injury led to interest in Zn^2+^ as a distinct ionic mediator of excitotoxic injury [5-7]. This neuronal Zn^2+^ accumulation appears to reflect a combination of presynaptic vesicular Zn^2+^ release with translocation into postsynaptic neurons, and mobilization of Zn^2+^ already within neurons from cytosolic buffers in response to oxidative stress and acidosis [7,8]. 

 Like Ca^2+^, Zn^2+^ can be taken up into mitochondria [9,10], with some studies suggesting that its effects on mitochondria may be far more potent than those of Ca^2+^ [7,8,11-13]. Indeed, a number of recent studies provide evidence that endogenous Zn^2+^ induces effects on mitochondrial function in both *in vitro* (hippocampal slice) [14] and *in vivo* models of brain ischemia [15,16]. Further highlighting the parallels between Zn^2+^ and Ca^2+^, modest Zn^2+^ exposures that do not induce rapid injury have still been found to induce NOX in cultured neurons, which can contribute to slowly evolving neurotoxicity [17,18]. 

 In light of above observations, the present study was undertaken to examine respective contributions of mitochondria and NOX to ROS generation in response to rapid Ca^2+^ or Zn^2+^ loading in cortical neuronal cultures. We find that each of these ions is taken up into mitochondria upon acute cytosolic loading. However, ROS generation following the acute Ca^2+^ loads appeared to derive from both NOX and mitochondria, whereas after Zn^2+^ loading, mitochondria appeared to be the dominant source of ROS. Furthermore, block of the mitochondrial Ca^2+^ uniporter (MCU), through which Zn^2+^ as well as Ca^2+^ can enter the mitochondrial matrix [9,13,19], blocked Zn^2+^ triggered ROS production, suggesting that much of the ROS generation occurs specifically in response to Zn^2+^ entry into mitochondria. Finally, studies were carried out in the presence of an oxidizing agent, 2,2'-dithiodipyridine (DTDP), which prevents Zn^2+^ binding to cytosolic metalloproteins like metallothioneins, in order to model the oxidative intracellular ischemic milieu, in which cytosolic Zn^2+^ buffering is impaired. Under these conditions, far lower Zn^2+^ exposures resulted in significant mitochondrial Zn^2+^ uptake and consequent ROS generation. These observations suggest that Ca^2+^ and Zn^2+^ can both trigger rapid ROS generation, but that Zn^2+^-dependent ROS generation occurs specifically after Zn^2+^ entry into mitochondria via the MCU, an effect that may be of particular importance during ischemia or other conditions in which cytosolic Zn^2+^ buffering is impaired. 

## Results

### NADPH oxidase contributes substantially to acute Ca^2+^- but not Zn^2+^-dependent ROS generation

Cortical neuronal cultures were prepared as described and allowed to mature for at least 10 days before use in experiments. To assess ROS generation, cultures were loaded with the superoxide-preferring indicator, HEt, which is oxidized by superoxide radicals into a highly fluorescent compound, ethidium [1]. Ethidium intercalates and binds to DNA resulting in amplification of fluorescence, providing high sensitivity and resulting in predominant visualization of the signal in the nucleus. Because the oxidized dye accumulates, the ROS production rate was assessed as the rate of fluorescence increase over time, and net ROS production was assessed as the increase in fluorescence (∆F) over baseline. To compare ROS generation across experiments, all fluorescence readings for a given cell (F_x_) were normalized to the average fluorescence of that cell during a 10 min baseline period (F_0_).

 Ca^2+^ loading was induced by exposure to NMDA (100 µM, 30 min) to strongly activate highly Ca^2+^-permeable NMDA-type glutamate channels, and was carried out in HEPES buffered salt solution containing physiological (1.8 mM) Ca^2+^, an exposure which predictably induces rapid and high rises in intracellular Ca^2+^ (estimated in the 10’s of µM) [20]. Zn^2+^ can enter neurons through a number of routes including voltage-gated Ca^2+^ channels (VGCC) or Ca^2+^-permeable α-amino-3-hydroxy-5-methyl-4-isoxazolepropionic acid (AMPA) channels [8]. However, Ca^2+^-permeable AMPA channels are only present on small minorities of neurons in the cultures [21], and VGCC do not permit sufficiently rapid Zn^2+^ entry to cause strong and rapid ROS generation in most neurons [22]. Therefore, to rapidly induce large Zn^2+^ loads in virtually all neurons, the cultures were pre-exposed to the membrane-permeable Zn^2+^ ionophore, pyrithione (10 µM) prior to a 5 min exposure to Zn^2+^ (300 µM) with pyrithione. Although most of our prior studies examining effects of Zn^2+^ entry into neurons on mitochondrial function were carried out with Ca^2+^ present in the extracellular buffer [10,22], in the present study, to fully discriminate effects of Zn^2+^ and Ca^2+^, all Zn^2+^ exposures were carried out in Ca^2+^-free buffer. 

**Figure 1:**
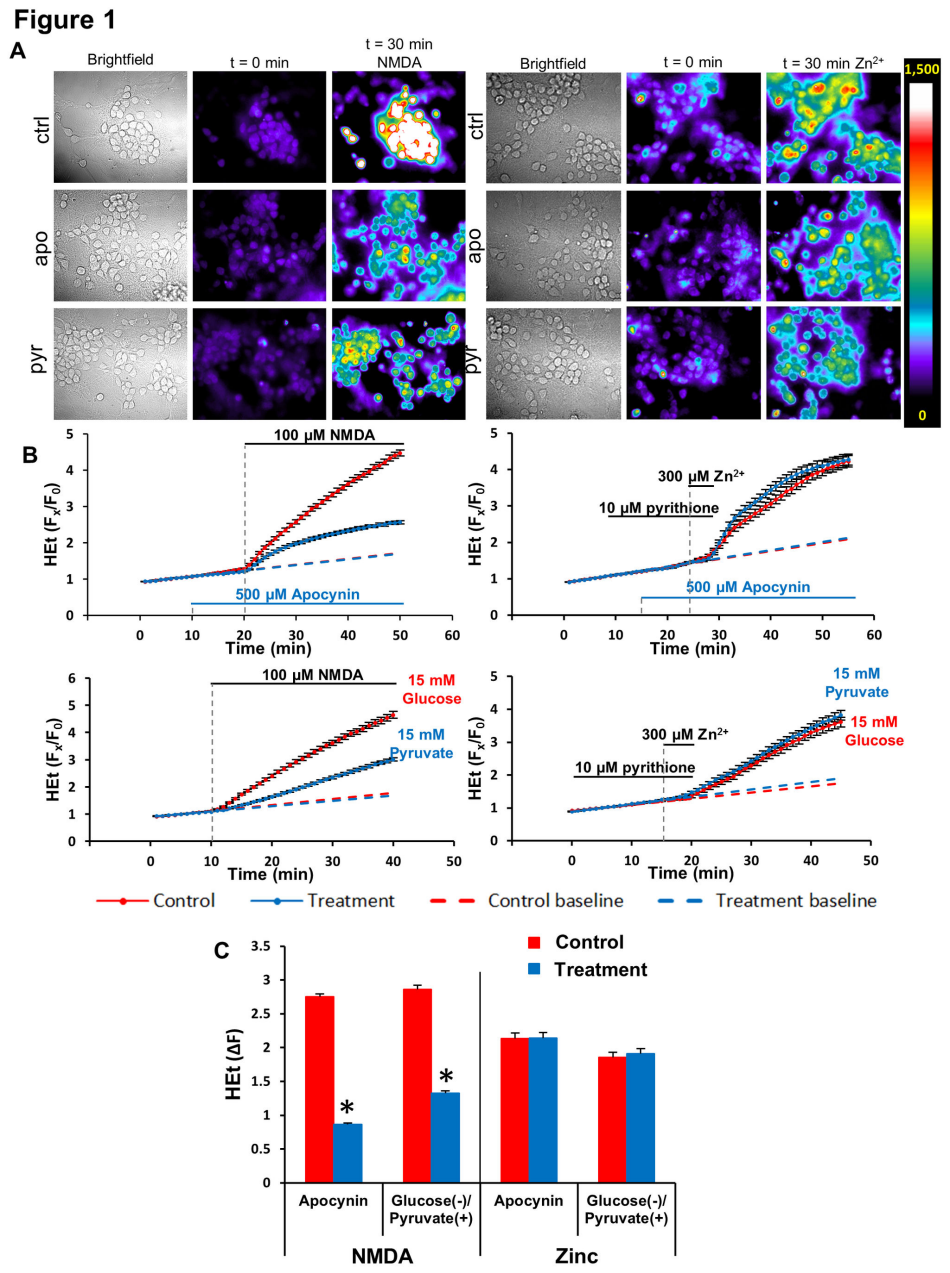
NADPH oxidase (NOX) inhibition attenuates acute Ca^2+^ - but not Zn^2+^-induced ROS production. HEt-loaded cultures were exposed to 100 μM NMDA (30 min) or 300 μM Zn^2+^/pyrithione (5 min) alone (red) or after pre-treatment with and in the presence of apocynin (500 µM), or in glucose-free media supplemented with pyruvate (15 mM) (blue) as described (see Materials and Methods). **A**: Representative images of selected fields of neuronal cultures (“Brightfield”), and pseudocolor images (400x) of HEt fluorescence from these neurons before and 30 min following onset of exposure to NMDA (left) or Zn^2+^/pyrithione (right). The pseudocolor bar shows the 12-bit fluorescence intensity range. **B**: Traces show time course of HEt ∆F, normalized to baseline values (F_x_/F_0_). Dashed lines show linear extrapolation of baseline. Traces show mean ± SD values from 4 experiments. **C**: Quantification of HEt ∆F changes. Values show F_x_/F_0_ increases after subtraction of the extrapolated baseline value, 30 min after onset of the exposure. ∆F values each represent means (± SEM) of the 4 experiments; * indicates difference from NMDA alone (p< 0.001) by 2-tailed t test.

 In the absence of either NMDA or Zn^2+^ addition, HEt fluorescence gradually increased in a linear fashion throughout the recording episode, presumably reflecting slow oxidation of the indicator due to basal metabolic activity of the cell (data not shown). With NMDA exposure, however, a sharp HEt ∆F began several min after the addition of the NMDA, with fluorescence increasing over baseline (F_x_/F_0_) typically ~ 5 fold within 30 min. The Zn^2+^ exposures caused a similar sharp HEt ∆F, which began typically 3-4 min after washout of the Zn^2+^/pyrithione (Figure 1A, B). 

 Two interventions were used to block the contribution of NOX to the ROS generation. First, we used the nonselective NOX inhibitor, apocynin (500 µM), which blocks the membrane translocation and activation of multiple NOX isoforms, including the primary neuronal isoform, NOX-2 [4,23,24]. In addition, we carried out some experiments, in which pyruvate (15 mM) was substituted for glucose as energy substrate (see Materials and Methods), since nicotinamide adenine dinucleotide phosphate (NADPH), the substrate of NOX, is primarily generated by the hexose monophosphate shunt during glycolysis. Both of these maneuvers substantially attenuated both the rate and extent of HEt oxidation induced by NMDA, while having no detectable effect on the Zn^2+^-triggered signal over the 30 min period following the start of the exposures (Figure 1A, B, C). Thus, these data suggest that NOX contributes importantly to acute ROS generation triggered by Ca^2+^ influx but does not contribute substantially to acute Zn^2+^-dependent ROS generation. 

### Mitochondria are the primary source of ROS generated after acute Zn^2+^ loads, but only account for a portion of the ROS induced by Ca^2+^ loading

**Figure 2:**
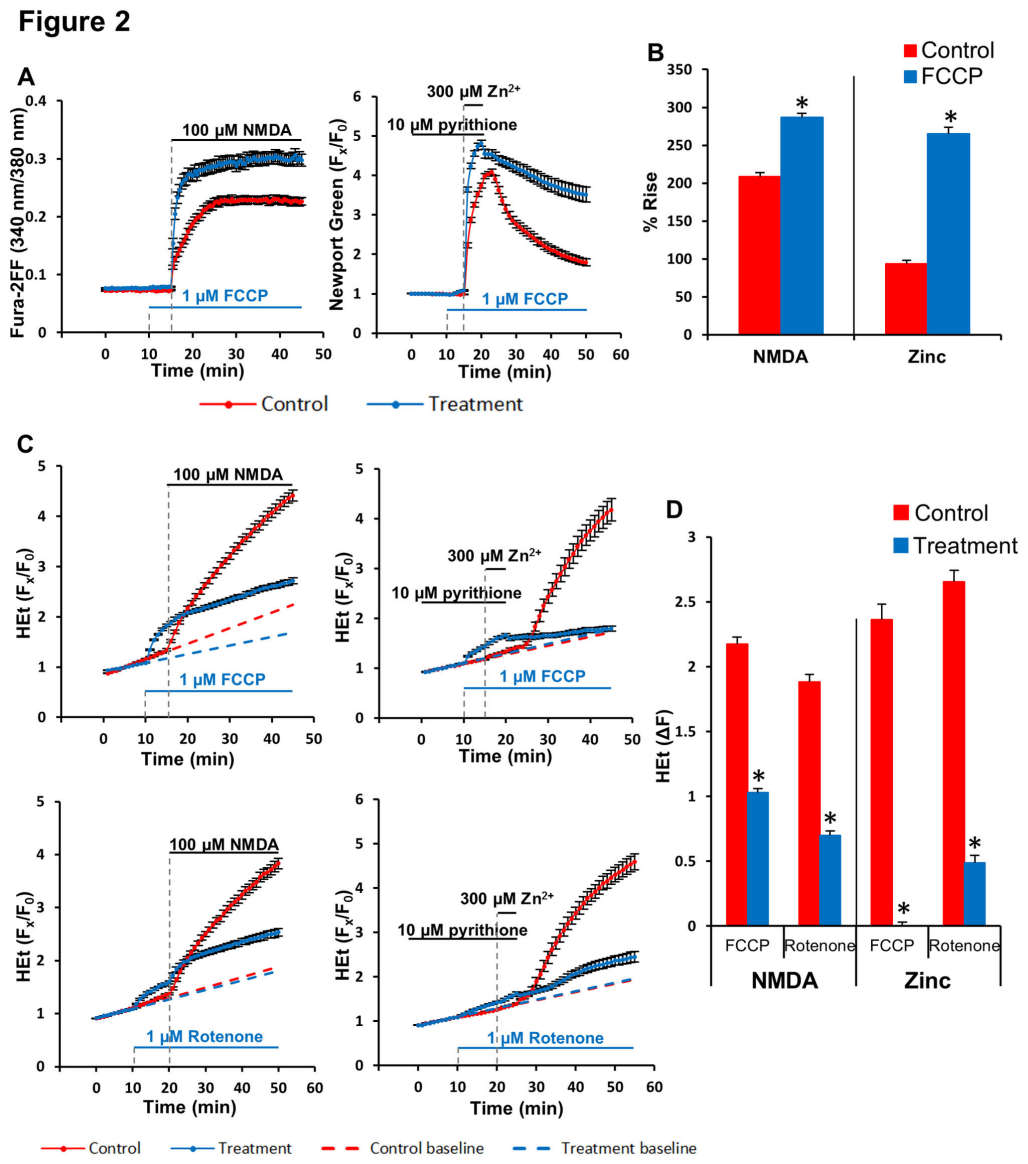
Effects of mitochondrial inhibition on cytosolic Ca^2+^ and Zn^2+^ rises and consequent ROS generation. Fura-2FF-, Newport Green-, or HEt-loaded cultures were exposed to 100 μM NMDA (30 min) or 300 μM Zn^2+^/pyrithione (5 min) alone (red) or after pre-treatment with and in the presence of FCCP (1 µM) or rotenone (1 µM) (blue) as described (see Materials and Methods). **A**: Time course of Ca^2+^ rises (assessed as Fura-2FF 340/380 nm fluorescence ratios) after NMDA exposure (left), and of Zn^2+^ rises (assessed as Newport Green F_x_/F_0_) after Zn^2+^/pyrithione exposure (right). Traces show mean ± SD values from 4 experiments. **B**: Quantification of ion-sensitive indicator (Fura-2FF or Newport Green) fluorescence changes. Values show % rise of the of the Fura2FF ratio (340 nm/380 nm) after NMDA exposure, or of Newport Green fluorescence after Zn^2+^/pyrithione exposure from baseline until 30 min after onset of exposure. Values represent means (± SEM) of the 4 experiments; * indicates difference from control condition (p< 0.05) by 2-tailed t test. **C**: Time course of ROS generation (assessed as HEt F_x_/F_0_), after exposures to NMDA (left) or to Zn^2+^/pyrithione (right). Dashed lines show linear extrapolation of baseline. Traces show mean ± SD values from 4 experiments. **D**: Quantification of HEt ∆F changes. Values show F_x_/F_0_ increases, after subtraction of expected increase in the absence of ion load (baseline extrapolated value), 30 min after onset of the exposure. Values represent means (± SEM) of the 4 experiments; * indicates difference from control condition (p< 0.01) by 2-tailed t test.

Mitochondria can buffer cytosolic Ca^2+^ and Zn^2+^ rises via electrogenically driven entry through the MCU into the matrix [10,25,26]. To examine the role of mitochondrial uptake on the magnitude of the acute Ca^2+^ and Zn^2+^ rises, we used the mitochondrial protonophore, carbonyl cyanide 4-(trifluoromethoxy)phenylhydrazone (FCCP) to dissipate the proton gradient across the inner membrane, thus eliminating the driving force for the uptake while inducing release of Ca^2+^ or Zn^2+^ ions already present in the mitochondria. To image the acute cytosolic Ca^2+^ or Zn^2+^ rises we used the low-affinity Ca^2+^ indicator, Fura-2FF (K_d_ ~ 25 µM; Teflab manual), and the low-affinity Zn^2+^ indicator, Newport Green (K_d_ ~ 1 µM; Life Technologies manual). When FCCP (1 µM) was added 5 min before acute 100 µM NMDA or 300 µM Zn^2+^/pyrithione exposures as above, the magnitude of the resultant cytosolic Ca^2+^ and Zn^2+^ rises were significantly increased, indicating that in control conditions (without FCCP) a substantial amount of either of these ions is taken up into mitochondria (Figure 2A,B).

 Next, to assess the contribution of mitochondria to the ROS generated in response to the Ca^2+^ or Zn^2+^ loads, HEt loaded cultures were pretreated with either FCCP (1 µM, 5 min), or with the complex I inhibitor, rotenone (1 µM, 10 min) prior to initiating the NMDA or Zn^2+^/pyrithione exposures. While both FCCP and rotenone by themselves induced a small increase in HEt fluorescence (see Figure 2C, during pretreatment episodes, prior to initiating the NMDA or Zn^2+^/pyrithione exposures), they both largely eliminated the acute Zn^2+^ triggered HEt signal, but only partially attenuated the Ca^2+^ triggered signal (Figure 2C,D). 

### Zn^2+^ dependent ROS generation is specifically linked to Zn^2+^ uptake into mitochondria via the MCU

 The observation that cytosolic Zn^2+^ loads appear to trigger ROS generation of mitochondrial origin does not necessarily mean that the Zn^2+^ acts within the mitochondria. However, present observations that treatment with FCCP not only depolarizes mitochondria and diminishes their Zn^2+^ uptake but also markedly decreases the resultant ROS production, is highly suggestive of an intramitochondrial site of the Zn^2+^ effects. 

**Figure 3:**
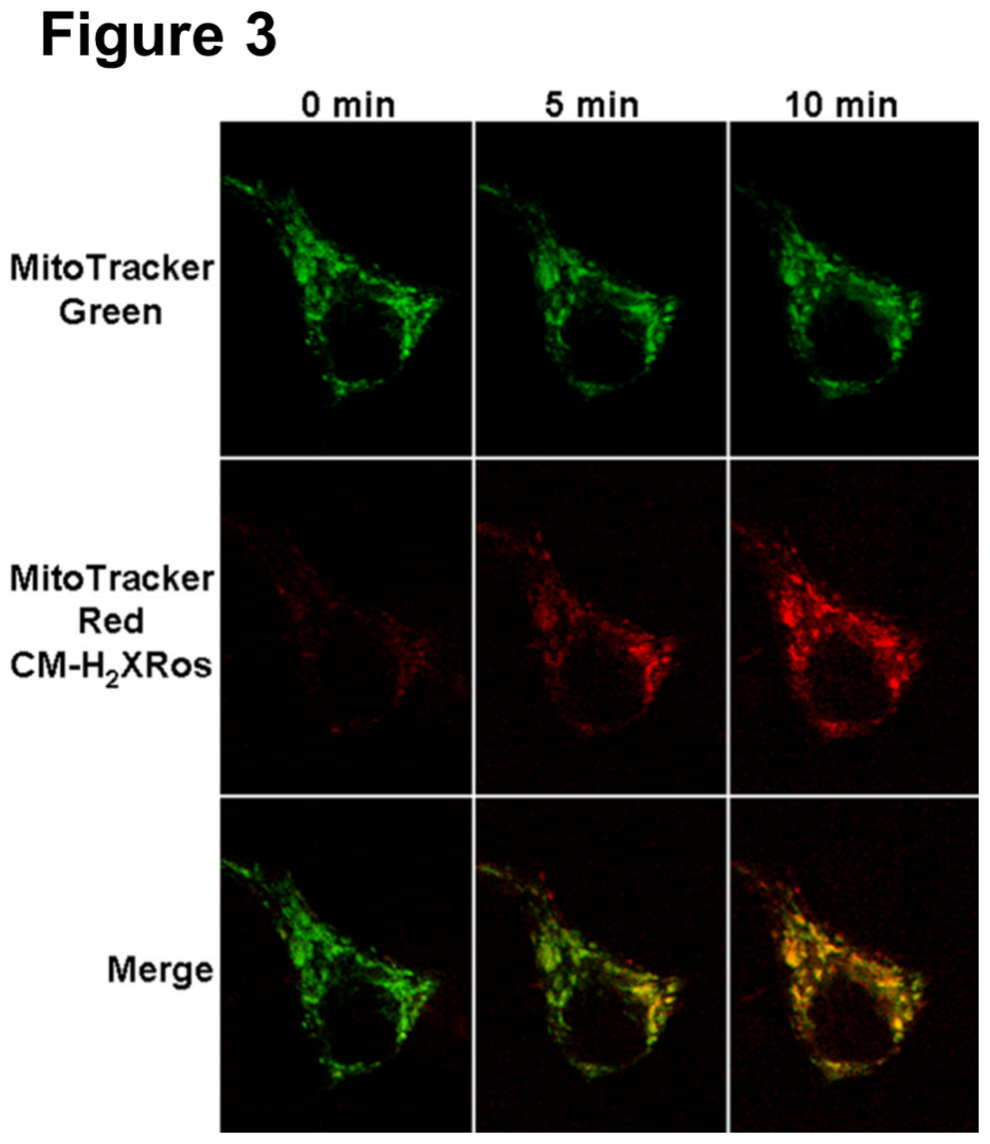
Mitochondrial localization of Zn^2+^ induced ROS production. Cultures were co-loaded with MitoTracker Green and MitoTracker Red CM-H_2_XRos (MTR-CMH_2_) and confocal images (1000x) obtained prior to Zn^2+^ treatment (t = 0 min), at the end of the 5 min 300 μM Zn^2+^/pyrithione exposure (t = 5 min), and 5 min after washout (t = 10 min). Note the marked increase in MTR-CMH_2_ fluorescence that co-localizes with MitoTracker Green, strongly suggestive of a mitochondrial site of ROS production.

 As discussed above, despite its sensitivity, HEt fluorescence signals do not provide information about the site of the ROS generation, as the fluorescence of the oxidized product is greatest in the nucleus, where interactions with DNA amplifies its emission. In order to obtain more information on the origin of the ROS produced by Zn^2+^ loads, we made use of a different indicator, MitoTracker Red CM-H_2_XRos (MTR-CMH_2_), which has previously been found to provide sensitive and specific visualization of mitochondrial ROS generation [27]. MTR-CMH_2_, a derivative of X-rosamine, accumulates in active polarized mitochondria, is oxidized by ROS into the fluorescent form, and is retained in mitochondria upon depolarization. Cultures were co-loaded with this indicator along with the fluorescent mitochondrial marker, MitoTracker Green (see Materials and Methods). For these studies a confocal microscope (Olympus IX70 with Bio-Rad Radiance 2000 Laser Scanning System) was used to aid in the visualization of signal emanating from individual mitochondria, and high-resolution images obtained before, at the end of a 5 min exposure to 300 µM Zn^2+^/10 µM pyrithione, and again 5 min after washout. Under these conditions, discrete foci of increasing red fluorescence were seen 2-3 min after the addition of the Zn^2+^, which co-localized with MitoTracker Green (Figure 3), further supporting a mitochondrial origin of the Zn^2+^-triggered ROS generation.

**Figure 4:**
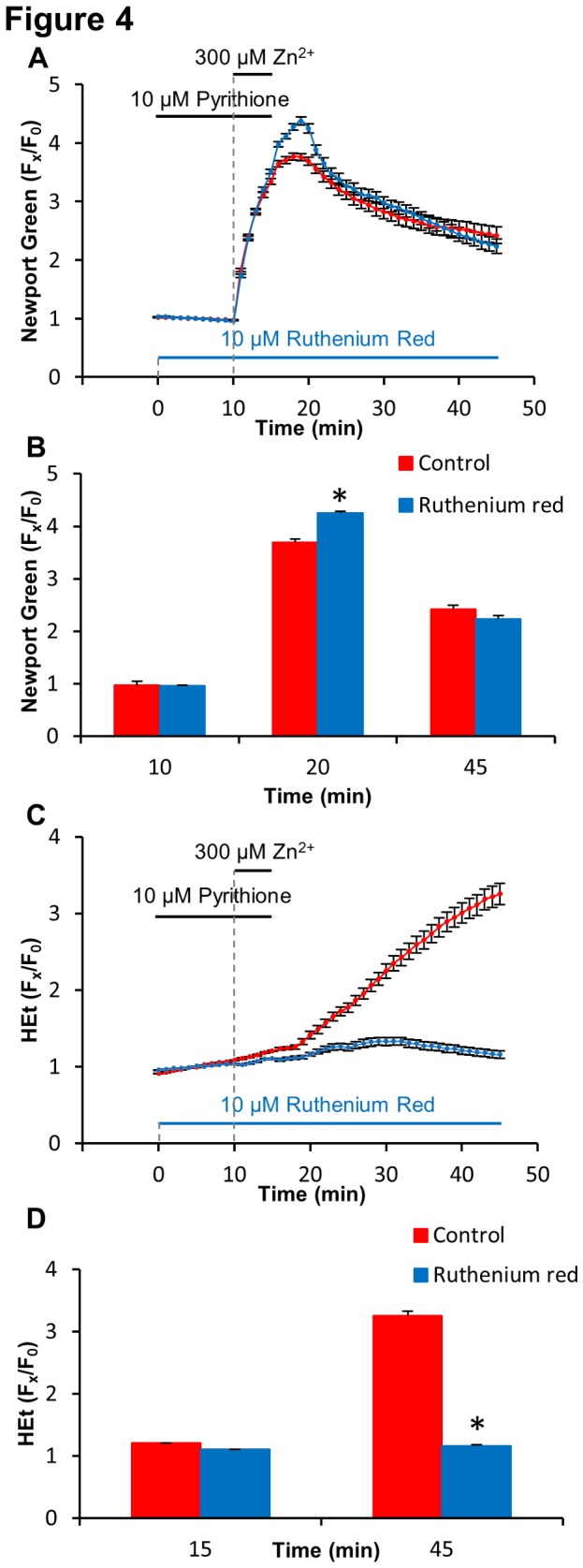
Blockade of the MCU increases cytosolic Zn^2+^ rises while attenuating consequent ROS production. Newport Green or HEt-loaded cultures were exposed to 300 μM Zn^2+^/pyrithione for 5 min alone (red) or after pre-treatment with and in the presence of RR (10 µM) (blue) as described (see Materials and Methods). **A**: Time course of Zn^2+^ rises (assessed as Newport Green F_x_/F_0_). Traces show mean ± SD values from 4 experiments. **B**: Quantification of Newport Green fluorescence changes. Values show Newport Green fluorescence changes ( F_x_/F_0_) at the times indicated on the traces shown in A, above. Values represent means (± SEM) of the 4 experiments; * indicates difference from control condition (p< 0.01) by 2-tailed t test. **C**: Time course of ROS generation (assessed as HEt F_x_/F_0_). Traces show mean ± SD values from 4 experiments. **D**: Quantification of HEt fluorescence changes. Values show HEt fluorescence changes ( F_x_/F_0_) at the times indicated on the traces shown in C, above. Values represent means (± SEM) of the 4 experiments; * indicates difference from control condition (p< 0.01) by 2-tailed t test.

 Next, to further assess the dependence of Zn^2+^-triggered mitochondrial ROS generation upon specific Zn^2+^ entry into these organelles, we attempted to examine effects of pharmacological block of the Zn^2+^ entry on ROS generation. Zn^2+^ appears to enter mitochondria through the MCU, and in past studies, the MCU blocker ruthenium red (RR) has been found to inhibit Zn^2+^ uptake into isolated mitochondria [9,28], and antagonize downstream effects including mitochondrial depolarization and swelling [13,29]. As these prior studies were carried out in isolated mitochondria, however, we first sought to further confirm the ability of RR to penetrate the neurons and antagonize Zn^2+^ entry into mitochondria by assessing its effects on the magnitude of the acute Zn^2+^ rises. Cultures were loaded with Newport Green and exposed to 300 µM Zn^2+^/pyrithione as above, either with or without pre-exposure to RR (10 µM). As previously observed with FCCP, the RR pre-treatment resulted in a significant increase in the peak level of the cytosolic Zn^2+^ rise, strongly suggesting that RR antagonizes Zn^2+^ buffering by mitochondria (mean % increase ± SEM in 4 expts of 385±11.4 with Zn^2+^/pyrithione alone vs 452±5.6 with RR, p< 0.01; Figure 4). Next, we used HEt-loaded cultures to examine effects of RR on the Zn^2+^-triggered ROS generation, and found it to substantially attenuate the HEt ∆F, similarly to FCCP or rotenone. This provides new support for an intramitochondrial site from which Zn^2+^ exerts its effects to trigger the acute ROS generation. 

### Oxidative disruption of cytosolic Zn^2+^ buffering dramatically increases the potency through which Zn^2+^ loads impact mitochondria

 The intense 300 µM Zn^2+^/pyrithione exposures, while useful to illustrate potential effects of very strong acute cytosolic Zn^2+^ rises, are of uncertain relevance to events that may occur *in vivo* in conditions like ischemia, as the magnitude of intracellular Zn^2+^ loading achieved may be greater than that readily induced upon mobilization of endogenous Zn^2+^ pools and stores. Neurons contain substantial cytosolic Zn^2+^ buffering capacity, much of which may be due to the presence of the metallothioneins or other Zn^2+^ binding metalloproteins [8]. Zn^2+^ binding to metallothioneins is destabilized under conditions of oxidative stress and acidosis [30,31], both of which occur prominently during ischemia. Indeed, metallothioneins appear able to mediate complex and disparate effects on Zn^2+^-dependent neurotoxicity depending on precise conditions, providing protection by buffering Zn^2+^ loads and serving as a source from which injurious Zn^2+^ can be mobilized under conditions of oxidative stress [19,32]. Indeed, it appears that oxidant-sensitive cytosolic Zn^2+^ pools and mitochondria can both sequester Zn^2+^, with release from one of them resulting in transfer of Zn^2+^ to the other [25]. The sulfhydryl-oxidizing agent, DTDP, has often been used to release Zn^2+^ from and prevent Zn^2+^ binding to cytosolic Zn^2+^ binding proteins, causing increases in free cytosolic Zn^2+^ levels in cultured neurons [33]; therefore, we used DTDP exposure to mimic the impaired cytosolic buffering that likely occurs during brain ischemia. 

**Figure 5:**
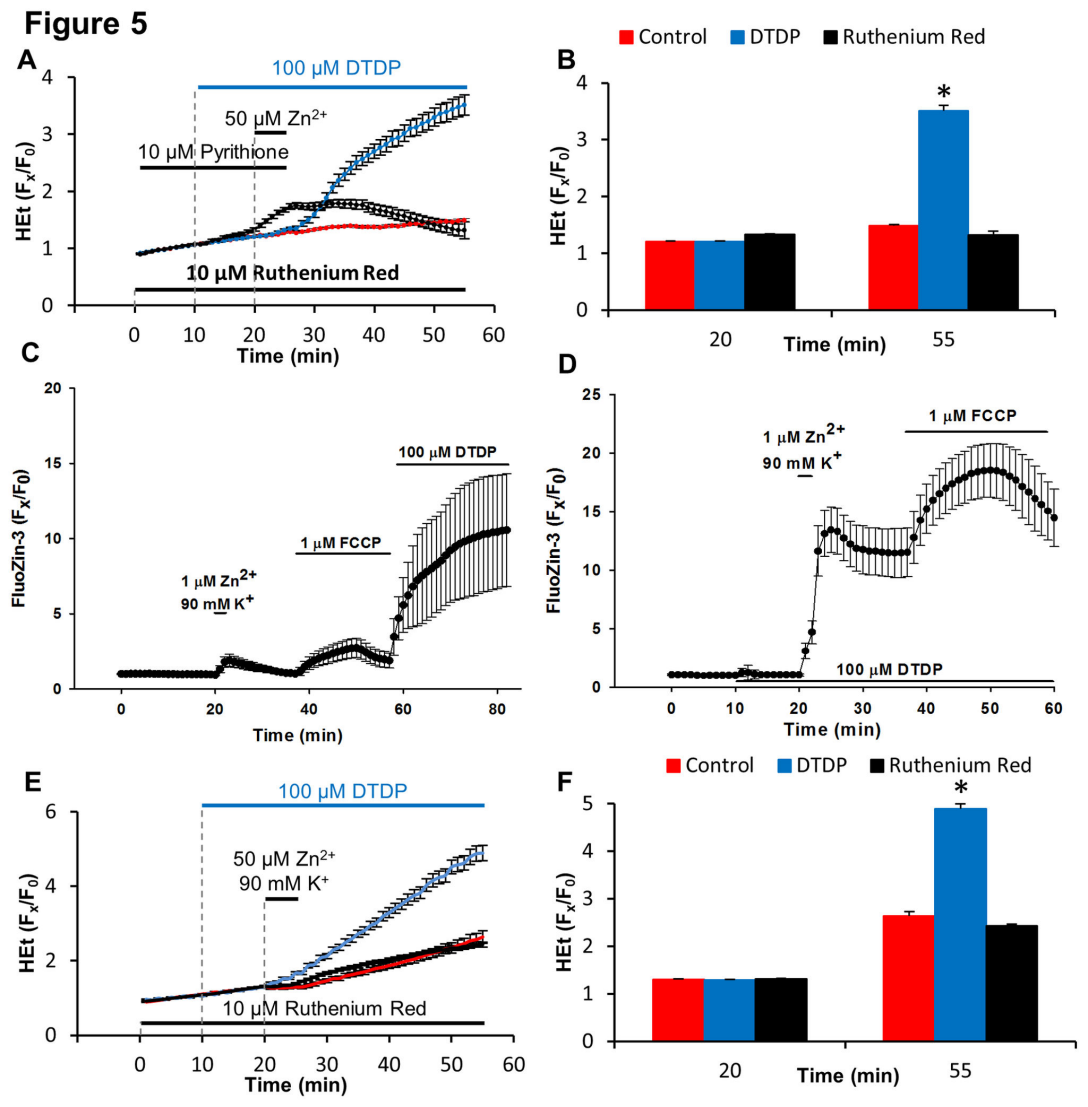
Under conditions of oxidative stress and disrupted Zn^2+^ buffering, lower levels of Zn^2+^ influx result in mitochondrial Zn^2+^ entry and ROS production. **A**: Effect of DTDP on Zn^2+^-triggered ROS generation. HEt-loaded cultures were exposed to 50 μM Zn^2+^/pyrithione alone (red), in the presence of DTDP (100 µM, blue), or with both DTDP and RR (10 µM, black) as indicated. Note that the Zn^2+^/pyrithione exposure only induced ROS generation in the presence of DTDP, and that the ROS production was eliminated by RR. Traces show mean ± SD values from 4 experiments. **B**: Quantification of HEt fluorescence changes. Values show HEt fluorescence changes (F_x_/F_0_) at the times indicated on the traces shown in A. Values represent means (± SEM) of the 4 experiments; * indicates difference from control condition (p< 0.01) by 2-tailed t test. **C**, **D**: Disruption of cytosolic Zn^2+^ buffering by DTDP markedly increases cytosolic Zn^2+^ rises and uptake into mitochondria. Fluo-Zin3-loaded cultures were exposed to 1 μM Zn^2+^ with 90 mM K^+^ (“Zn^2+^/high-K^+^”, to trigger a low level of Zn^2+^ influx), to FCCP (1 µM), or to DTDP (100 µM) as indicated. In C, when the cultures were first exposed to Zn^2+^/high-K^+^ there was a very small FluoZin-3 ∆F, and subsequent FCCP exposure, to depolarize the mitochondria and release mitochondrially-sequestered Zn^2+^ into the cytosol, caused only a slight further increase. However, adding DTDP after the FCCP produced a large FluoZin-3 ∆F, suggesting that the Zn^2+^ entering during the Zn^2+^/high-K^+^ exposure had been largely buffered in the cytosol with little entering the mitochondria. In contrast (**D**), when the cultures were first exposed to DTDP (100 µM), there was a minimal FluoZin-3 ∆F, but when the DTDP exposure was followed by Zn^2+^/high-K^+^, the cytosolic ∆F was dramatically increased, and subsequent FCCP exposure resulted in a marked further ∆F, indicative of Zn^2+^ having accumulated within the mitochondria. Traces show mean ± SD values from 120 neurons from 4 experiments. **E**: 50 μM Zn^2+^/high-K^+^ exposure cause mitochondrial ROS production only in the presence of DTDP. HEt-loaded cultures were exposed to 50 μM Zn^2+^/90 mM K^+^ alone (red) or after pre-treatment with and in the presence of DTDP alone (100 µM, blue) or with RR (10 µM, black) as indicated. Traces represent time course of HEt ∆F, normalized to baseline values (F_x_/F_0_) and show mean ± SD values from 4 experiments. **F**: Quantification of HEt fluorescence changes. Values show HEt fluorescence changes (F_x_/F_0_) at the times indicated on the traces shown in E. Values represent means (± SEM) of the 4 experiments; * indicates difference from control condition (p< 0.01) by 2-tailed t test.

 We next sought to determine whether impeding cytosolic Zn^2+^ buffering with DTDP would permit lower levels of Zn^2+^ loading to induce mitochondrial ROS generation. When cultures were exposed to 50 µM Zn^2+^/pyrithione, in the absence of DTDP, there was no HEt ∆F over baseline. DTDP (100 µM) alone also caused no HEt ∆F (data not shown). However, when the Zn^2+^ load was carried out in the presence of DTDP (before and during the Zn^2+^ exposure), a sharp HEt ∆F occurred, similar to that induced by 300 µM Zn^2+^/pyrithione. To confirm that this ROS generation was dependent upon mitochondrial Zn^2+^ uptake, identical exposures were carried out after exposure to RR, which almost completely blocked the HEt ∆F (Figure 5A,B).

 Although pyrithione is useful for eliciting large Zn^2+^ loads into all neurons, a drawback of this approach is that it may be relatively non-physiologic, since pyrithione is membrane permeable and could directly facilitate Zn^2+^ entry into intracellular pools, including mitochondria, or Zn^2+^ exit from the cell. We therefore examined effects of triggering Zn^2+^ entry in a rapid and more physiologically relevant fashion, using brief exposures to high (90 mM) K^+^ buffer, which depolarizes neurons and causes opening of VGCC, through which Zn^2+^ can enter [34]. In prior studies, we found that 5 min high-K^+^ exposures with 300 µM Zn^2+^ failed to cause rapid HEt ∆F in most neurons (only causing such a response in the subpopulation of neurons that possessed large numbers of Ca^2+^ permeable AMPA channels, through which Zn^2+^ permeates more rapidly than through VGCC) [22]. 

 To examine intracellular Zn^2+^ dynamics after inducing low sub-toxic Zn^2+^ entry through VGCC, we used the relatively high affinity Zn^2+^ indicator, FluoZin-3 (K_d_ ~ 15 nM) [35]. Exposure to 1 µM Zn^2+^ in the presence of high-K^+^ caused a very small FluoZin-3 ∆F, and subsequent FCCP exposure (to depolarize the mitochondria and release mitochondrially-sequestered Zn^2+^ into the cytosol) caused only a slight further increase. However, adding DTDP after the FCCP produced a large FluoZin-3 ∆F, suggesting that the Zn^2+^ had been largely buffered in the cytosol with little entering the mitochondria (Figure 5C). When the culture was exposed to DTDP (100 µM) before the 1 µM Zn^2+^/high-K^+^ exposure, a very slight ∆F occurred. However, when this DTDP exposure was followed by the 1 µM Zn^2+^/high-K^+^ exposure, the cytosolic ∆F dramatically increased. Furthermore, subsequent FCCP exposure resulted in a marked further ∆F, indicative of Zn^2+^ having accumulated within the mitochondria (Figure 5D). Thus, impairment of cytosolic Zn^2+^ buffering dramatically increases the amount of Zn^2+^ entering mitochondria upon relatively low level Zn^2+^ entry into the cell. 

 Finally, we carried out one additional assessment of ROS generation, using high-K^+^ to trigger Zn^2+^ entry as above, in the presence of 50 µM Zn^2+^. As with 50 µM Zn^2+^/pyrithione exposures, 50 µM Zn^2+^/high-K^+^ exposures by themselves induced no HEt ∆F, but a distinct HEt ∆F occurred when the cultures were pre-exposed to DTDP, which, as in the case of the 50 µM Zn^2+^/pyrithione/DTDP exposures, was substantially blocked by RR (Figure 5E,F). Thus, these data lend support to the idea that under conditions of impaired cytosolic Zn^2+^ buffering as likely occurs as a result of oxidative stress and acidosis during brain ischemia, relatively low levels of Zn^2+^ entry may result in Zn^2+^ passage through the MCU into mitochondria at levels sufficient to induce acute mitochondrial dysfunction with excess ROS generation. 

## Discussion

### Summary of principal findings

 The present study seeks to compare acute ROS generation induced in cortical neurons by strong cytosolic Ca^2+^ loading with that induced by Zn^2+^ loading, events that are of likely relevance to neurodegeneration occurring in conditions of strong excitotoxic activation as occurs in ischemia or prolonged seizures. The rationale for the specific comparisons addressed by the studies (Ca^2+^ vs Zn^2+^; NOX vs mitochondria) reflect persistent uncertainties as to the key early events underlying excitotoxic neurodegeneration under differing circumstances. First, whereas early *in vitro* studies focused on Ca^2+^ overload as the key trigger of excitotoxic injury, subsequent studies in native tissue preparations (*in vivo* and slice models) found compelling evidence for important contributions of endogenous Zn^2+^ accumulation in triggering injury after ischemia or prolonged seizures. In addition, whereas imaging studies with “Ca^2+^ indicators” had long been interpreted as demonstrating Ca^2+^ transients and associated Ca^2+^ triggered effects, it is now known that Ca^2+^ indicators almost all bind and respond to Zn^2+^ with far higher affinity than Ca^2+^, raising the strong possibility that some effects previously attributed to Ca^2+^ might in fact be Zn^2+^-mediated [36]. Thus, as past *in vitro* studies have clearly demonstrated that cellular loading with either of these ions can trigger ROS generation [2,3,22], there is persistent uncertainty as to the likely dominant ionic “instigator” of excitotoxic injury in ischemia and prolonged seizures. 

 We find, in line with a recent study suggesting that Ca^2+^ causes rapid translocation and activation of NOX and consequent superoxide generation, that the Ca^2+^-dependent ROS signal was substantially (but not completely) NOX-dependent. In contrast, the Zn^2+^-triggered ROS signal was markedly diminished by mitochondrial inhibitors or by MCU inhibition, but appeared to be NOX-independent. As the strong Zn^2+^ exposures initially employed are of uncertain direct physiological relevance, we next examined effects of the sulfhydryl-oxidizing agent DTDP, which impairs Zn^2+^ binding to cytosolic Zn^2+^ buffering proteins, and thus reproduces deficiencies in cytosolic Zn^2+^ buffering likely caused by oxidative stress and acidosis during *in vivo* cerebral ischemia. Under these conditions, far lower Zn^2+^ loads resulted in substantial mitochondrial Zn^2+^ uptake and ROS generation, suggesting that during ischemia, physiological levels of Zn^2+^ accumulation might well enter mitochondria and induce deleterious effects on their function with release of ROS. 

### Ca^2+^ and excitotoxic neurodegeneration

 The key finding that removal of Ca^2+^ from the media during brief glutamate or NMDA exposures to cultured neurons markedly diminished the injury that evolved over the subsequent hours has implicated Ca^2+^ entry through NMDA channels as a critical trigger of excitotoxic neurodegeneration. Consequently, it was predicted that NMDA receptor blockade would have potent protective effects against ischemic neurodegeneration [37], and a large number of studies have examined mechanisms of this Ca^2+^-dependent injury. 

 One target of the Ca^2+^ effects is mitochondria, which clearly can buffer large Ca^2+^ loads [26,38,39]. Furthermore, a number of studies concluded that rapid Ca^2+^ uptake into mitochondria caused ROS generation [1-3]. However, effects of Ca^2+^ on mitochondria are complex and mechanisms through which it increases mitochondrial ROS release are poorly understood and depend critically upon the paradigm employed. Mechanisms that have been suggested to be involved include stimulation of certain mitochondrial dehydrogenases, respiratory inhibition at complex I or III, and mitochondrial permeability transition pore induction, with consequent direct release of ROS, loss of cytochrome c, or loss of intramitochondrial antioxidant enzymes and glutathione [40-42].

 Further studies have highlighted extramitochondrial routes through which cellular Ca^2+^ loading might trigger excitotoxic injury. One of these is the activation of nitric oxide synthetase (NOS), a free radical generating enzyme, which is linked intracellularly to the NMDA receptor complex. Interestingly, for this injury pathway, the critical factor does not appear to be the amount of Ca^2+^ entering the neuron, but rather the specific activation and passage of Ca^2+^ through NMDA channels [43]. Other studies have highlighted Ca^2+^-dependent NOX activation via a pathway involving phosphoinositide 3-kinase and protein kinase c-zeta activation [4,44]. Furthermore, NOX activation during *in vivo* ischemia, driven in part by high glucose [45], appears to contribute to degeneration after stroke [46]. Both NOS and NOX appear to mediate degeneration in part via a pathway in which oxidative DNA damage causes activation of the DNA repair enzyme, poly(ADP-ribose) polymerase-1 (PARP), resulting in depletion of NAD^+^ and ATP [47].

### Zn^2+^ and excitotoxic neurodegeneration

In light of evidence that neuronal Zn^2+^ accumulation contributes to neurodegeneration in disease conditions, a large number of studies over the past two decades have examined relevant mechanisms. One key question concerned the source of the injurious neuronal Zn^2+^ accumulation. One source appears to be presynaptic vesicular Zn^2+^, which is co-released with glutamate from presynaptic terminals and enters post-synaptic neurons through routes, including VGCC and Ca^2+^-permeable AMPA channels [22,34,48]. However, it has become apparent that Zn^2+^ does not need to enter neurons from outside in order to injure them. There are large pools of Zn^2+^ within neurons bound to buffering proteins like metallothioneins. Zn^2+^ binding to these proteins is sensitive to both pH and oxidative stress [30,31], and these metabolic changes occur prominently in pathological conditions of ischemia or prolonged seizures, likely contributing to free Zn^2+^ accumulation in these conditions. Of note, even cultured neurons contain sufficient oxidant-releasable Zn^2+^ stores to induce injury, as exposure to the sulfhydryl-oxidizing agent DTDP induced both cytosolic Zn^2+^ rises and Zn^2+^-dependent injury [33], and it is apparent that these intracellular Zn^2+^ pools can contribute to neurodegeneration *in vivo* as well [32,49].- 

 Subsequent studies concerned mechanisms of Zn^2+^ toxicity. Interestingly, a number of studies indicated prominent parallels with Ca^2+^. Early studies found that Zn^2+^ exposures less intense than those used in this study to induce acute ROS generation still caused slowly evolving neurodegeneration with oxidative features [50], resulting in part from protein kinase C-dependent induction and activation of NOX [18]. Further studies indicated that this NOX activation, along with induction of NOS, resulted in activation of PARP, NAD^+^ and ATP depletion and cell death [17], much as in the case of Ca^2+^-dependent excitotoxicity. Thus, the role of NOX in more slowly developing Zn^2+^-dependent neurotoxicity is in contrast to present findings that did not show evidence of NOX contribution in acute Zn^2+^-triggered ROS production. 

 In other studies we correlated Zn^2+^ rises after entry through different routes with ROS generation, and found that rapid Zn^2+^ entry through Ca^2+^-permeable AMPA channels appeared to cause acute mitochondrial ROS generation that correlated with potent induction of toxicity [22]. Further studies showed that Zn^2+^ was taken up into mitochondria, causing depolarization and swelling in a MCU dependent fashion, and that it induced swelling of isolated mitochondria with far greater potency than Ca^2+^ [10,13,29]. 

 Whereas Zn^2+^ was long ago found to inhibit electron transport [51], more recent studies have suggested mechanisms of its effects. Like Ca^2+^, it can enter mitochondria through the MCU [9], can antagonize the bc1 center of the electron transport chain [52], and appears, unlike Ca^2+^, to potently and irreversibly inhibit major enzymes of mitochondrial energy production and antioxidant defense [12,53], triggering induction of the mitochondrial permeability transition pore [12,13,54]. 

### Possible relevance to disease

 Whereas studies described above regarding Zn^2+^ effects on mitochondria all employ addition of exogenous Zn^2+^, recent studies provide strong support to the idea that mobilization of endogenous Zn^2+^ in pathological conditions yields levels sufficient to induce mitochondrial dysfunction. Specifically, recent studies suggest that endogenous Zn^2+^ contributes to mitochondrial dysfunction after *in vivo* ischemia [15,16]. Also, in hippocampal slices subjected to an oxygen glucose deprivation model of acute ischemia, we found that Zn^2+^ rises in CA1 neurons preceded and contributed to the occurrence of both irreversible mitochondrial depolarization and to the onset of a terminal Ca^2+^ deregulation [14]. 

 In summary, as time passes, the “landscape” of excitotoxic contributions in conditions like ischemia is growing progressively complicated, and the early focus on NMDA receptor-mediated Ca^2+^ overload is clearly too limited. Contributions of Zn^2+^ may have been underestimated for a number of reasons, including the presumption that signals from “Ca^2+^ indicators” were all due to Ca^2+^, lack of awareness of the degree to which metabolic derangements in these disease conditions may promote intracellular Zn^2+^ accumulation, and the primary focus of disease studies on delayed events associated with “reperfusion” that paid inadequate attention to early and possibly highly impactful events occurring during acute ischemia. It is most likely that, the true evolution of “excitotoxic” injury after ischemia involves a host of events, some Ca^2+^- and some Zn^2+^- initiated, and some occurring acutely (perhaps including mitochondrial dysfunction), and, if the neurons survive the acute insult, others, likely including activation of NOX and downstream injury pathways, playing roles in the determination of cell fate over hours to days after “reperfusion”. 

 In light of recent evidence for early Zn^2+^-dependent mitochondrial dysfunction in ischemia, present observations lend support to the idea that under conditions of impaired cytosolic Zn^2+^ buffering, as occurs in ischemia, physiologically relevant levels of Zn^2+^ entry and release from cytosolic buffers likely result in Zn^2+^ passage into mitochondria via the MCU. Present findings, together with emerging indications of the high potency of Zn^2+^ effects on mitochondria, lead us to suggest the hypothesis that such early mitochondrial Zn^2+^ accumulation provides a potent assault on mitochondrial function with an accompanying burst of ROS generation. The ROS could further destabilize cellular Zn^2+^ buffering, resulting in a cascading positive feedback cycle leading to mitochondrial disruption. Understanding and prevention of such a cycle in the early stages of ischemia may markedly increase prospects for neuronal survival beyond reperfusion into the post-ischemic period. 

## Materials and Methods

### Ethics Statement

 This study was carried out in strict accordance with the recommendations in the Guide for the Care and Use of Laboratory Animals of the National Institutes of Health. The protocol was approved by the Institutional Animal Care and Use Committee of the University of California, Irvine (Protocol Number: 1997-1267). All procedures were terminal and all animals were deeply anesthetized before starting any procedures to minimize suffering.

### Chemicals and Reagents

 Hydroethidine (HEt) was purchased from Assay Biotech (Sunnyvale, CA). Newport Green, FluoZin-3, AM, MitoTracker Green, MitoTracker Red CM-H_2_XRos, Pluronic F-127, tissue culture media and horse serum were purchased from Life Technologies (Grand Island, NY). Fura-2FF was obtained from Teflabs (Austin, TX). NMDA, rotenone, pyrithione, DTDP, ruthenium red and fetal bovine serum were purchased from Sigma-Aldrich (St. Louis, MO). FCCP was purchased from Tocris Bioscience (Ellisville, MO) and apocynin was obtained from Acros Organics (Morris Plains, NJ). All other chemicals and reagents were purchased from common commercial sources. 

### Cortical Cultures

 Cultures were prepared generally as previously described [21]. Briefly, dissociated mixed neocortical cell suspensions were prepared from 15-16 day CD-1 mouse embryos and plated on previously established astrocytic monolayers in glass-bottomed dishes, in media consisting of Eagle's Minimum Essential Medium (EMEM; Earle's salts prepared glutamine-free) supplemented with 10% heat-inactivated horse serum, 10% fetal bovine serum, 2 mM glutamine, and 25 mM glucose, and kept in a 37°C/5% CO2 incubator. After 3-4 DIV (days in vitro), non-neuronal cell division was halted by exposure to 10 μM cytosine arabinoside for 24 h, and the cultures were switched to an identical maintenance medium lacking fetal serum. The same procedure was used to prepare glial cultures, except that tissue was obtained from early postnatal (1–3 d) mice, media was supplemented with epidermal growth factor (10 ng/ml), and cell suspensions were plated directly on the polylysine- and laminin-coated coverslips. 

### Imaging Studies

 10–13 DIV cultures were mounted on the stage of a Nikon Diaphot inverted microscope equipped with a 75 W xenon lamp, a computer-controlled filter wheel, a 40 X 1.3 numerical aperture epifluorescence oil-immersion objective along with a green FITC fluorescence cube (Ex: 480 nm, dichroic: 505 nm, Em: 535 nm), a red TRITC fluorescence cube (Ex: 540 nm, dichroic: 565 nm, Em: 605 nm), and an ultraviolet Fura-2 fluorescence cube (Ex: NA, dichroic: 400 nm, Em: 510 nm). Emitted signals were acquired with a Sensys Photometrics intensified charge-coupled device camera and digitized by using MetaFluor Version 7.0 software (Molecular Devices LLC, Sunnyvale, CA). Background fluorescence was subtracted from images at the beginning of each experiment and all experiments were carried out at room temperature in HEPES-buffered medium (HSS), consisting of (in mM): 120 NaCl, 5.4 KCl, 0.8 MgCl_2_, 20 Hepes, 15 glucose, 1.8 CaCl_2_, 10 NaOH, pH 7.4. Camera gain and exposure were adjusted to give baseline maximal fluorescence levels of 200-300 arbitrary units of a maximal 12-bit signal output of 4,096 for all fluorophores. 

 ROS generation was assessed by examining fluorescence changes in cells loaded with the oxidation sensitive dye HEt (Ex: 510-560; Em: >590), using the red fluorescence cube. For the NMDA studies, cultures were loaded in the dark at room temperature with 5 μM HEt in HSS for 45 min and then washed into a static bath of HSS containing 5 μM HEt for the 10 min baseline recording. For the Zn^2+^ experiments, treatments were the same except that all exposures were in Ca^2+^-free HSS. HEt fluorescence measurements for each cell (F_x_) were normalized to the average fluorescence intensity for that cell during a 10 min period of baseline recording (F_0_).

 For pharmacological treatments, ruthenium red (RR, 10 μM), was added during the HEt loading, pyrithione (10 μM) was added at the onset of baseline recording and maintained through the Zn^2+^ exposure, and other drugs (500 μM apocynin, 1 μM FCCP, 1 μM rotenone, or 100 μM DTDP) were added prior to the NMDA (100 μM, 30 min) or Zn^2+^ (with 10 μM pyrithione or 90 mM K^+^, 5 min) exposures, and maintained throughout the duration of the recordings, as indicated. For pyruvate treatment, cells were switched to glucose-free HCSS containing 15 mM pyruvate during the HEt loading and maintained in this media throughout the imaging run. Control experiments showed that in the absence of NMDA or Zn^2+^ treatment, HEt fluorescence slowly increased in a linear fashion over the 60 min recordings. We thus obtained linear regressions of the 10 min baseline for each set of data, enabling comparison of endpoint fluorescence with expected values in the absence of Ca^2+^ or Zn^2+^ loading. 

 For [Zn^2+^]_i_ and [Ca^2+^]_i_ imaging, cultures were loaded in the dark with 5 μM of either the relatively low-affinity Zn^2+^ indicator, Newport Green diacetate (K_d_ ~ 1 µM, Ex: 490 nm, Em: 530 nm), the high affinity Zn^2+^ indicator, FluoZin-3 (K_d_ ~ 15 nM, Ex: 494 nm, Em: 516 nm), or the low-affinity Ca^2+^ indicator, Fura-2FF (K_d_ ~ 25 µM, Ex: 340-380 nm, Em: 510 nm) in HSS containing 0.2 % Pluronic F-127 and 1.5 % dimethlyl sulfoxide (DMSO) for 30 min at 25°C, then washed into HSS for Ca^2+^ imaging or Ca^2+^-free HSS for Zn^2+^ imaging, and kept in the dark for an additional 30 min. For Newport Green and FluoZin-3, the green fluorescence cube was used, while for Fura-2FF, excitation was via the filter wheel (340 and 380 nm filters), and emission via the Fura-2 filter cube. Experiments were carried out in a static bath of HSS or Ca^2+^-free HSS and drugs were added to the buffer for pre-treatments as indicated. For intracellular Zn^2+^, fluorescence measurements for each cell (F_x_) were normalized to the average fluorescence intensity for that cell during the first 10 min of the experiment (F_0_). Changes in intracellular Ca^2+^ were assessed as the ratio of the emission intensity (at 510 nm) when excited at 340 nm to that upon excitation at 380 nm (“340/380 emission ratio”). 

 Confocal microscopy of live dissociated cell culture was performed using an Olympus IX70 microscope equipped with a Bio-Rad Radiance 2000 laser scanning system. Images were collected with a 100 X oil-immersion objective using Zeiss LaserSharp 2000 software. Briefly, cells were loaded with 200 nM MitoTracker green (Ex: 490 nm, Em: 516 nm) and 500 nM MitoTracker Red CM-H_2_XRos (MTR-CMH_2_) (Ex: 579 nm, Em: 599 nm) for 30 min at 37°C in the dark, and then switched to 0 Ca^2+^ HCSS and 10 µM pyrithione for the imaging experiment using the confocal. An image was acquired prior to Zn^2+^ treatment, immediately after a 5 min exposure to 300 µM Zn^2+^/pyrithione, and again 5 min after washout. Images were scanned sequentially with a 488 nm line Argon laser for MitoTracker Green followed by a 543 nm line Green HeNe laser for MTR-CMH_2_. 

### Statistical Analysis

 Traces (with the exception of Figure 5 C, D) all represent the mean values from 4 independent experiments. In each experimental condition in each experiment a field containing many healthy appearing neurons was selected for imaging, and ~ 25-35 healthy-appearing neurons were prospectively chosen and marked for fluorescence measurements; after the exposure the fluorescence changes in these 25-35 cells was averaged to yield a single value constituting one independent replication of one condition in the experiment. In addition, we always carried out paired examinations of control or treatment exposures using sister cultures from the same preparation and studied on the same day. All compiled data are derived from at least 4 fully independent experiments, and statistics are based upon the number of independent experimental repetitions rather than the total numbers of cells studied for each condition in order to provide the most rigorous and conservative test of differences. Error bars on traces show means ± SD for the 4 experiments (or for 120 neurons from 4 dishes studied in 5 C, D). The bar graphs display the mean values of 4 experiments (± SEM), and significance values are based on n=4 for each condition. All significance values are based upon 2-tailed t-tests.
